# Assessing Treatment Success or Failure as an Outcome in Randomised Clinical Trials of COPD Exacerbations. A Meta-Epidemiological Study

**DOI:** 10.3390/biomedicines9121837

**Published:** 2021-12-05

**Authors:** Alexander G. Mathioudakis, Sachin Ananth, Thomas Bradbury, Balazs Csoma, Pradeesh Sivapalan, Elizabeth Stovold, Gustavo Fernandez-Romero, Zsofia Lazar, Gerard J. Criner, Christine Jenkins, Alberto Papi, Jens-Ulrik Jensen, Jørgen Vestbo

**Affiliations:** 1 Division of Infection, Immunity and Respiratory Medicine, School of Biological Sciences, The University of Manchester, Manchester M23 9LT, UK; Jorgen.Vestbo@Manchester.ac.uk; 2 North West Lung Centre, Wythenshawe Hospital, Manchester University NHS Foundation Trust, Manchester Academic Health Science Centre, Manchester M23 9LT, UK; 3 West Hertfordshire Hospital NHS Trust, Watford WD18 0HB, UK; sachin.ananth@doctors.org.uk; 4 The George Institute for Global Health, University of New South Wales, Sydney 1466, Australia; tbradbury@georgeinstitute.org.au (T.B.); christine.jenkins@sydney.edu.au (C.J.); 5 Department of Pulmonology, Faculty of Medicine, Semmelweis University, 1085 Budapest, Hungary; csomabalazs1@gmail.com (B.C.); zsofia.lazar@yahoo.com (Z.L.); 6 Section of Respiratory Medicine, Department of Internal Medicine, Herlev-Gentofte Hospital, 2900 Hellerup, Denmark; pradeesh.sivapalan.02@regionh.dk (P.S.); jens.ulrik.jensen@regionh.dk (J.-U.J.); 7 Department of Internal Medicine, Zealand University Hospital, 4000 Roskilde, Denmark; 8 Cochrane Airways Group, Population Health Research Institute, St George’s University of London, London SW17 0RE, UK; estovold@sgul.ac.uk; 9 Department of Thoracic Medicine and Surgery, Lewis Katz School of Medicine at Temple University, Philadelphia, PA 19140, USA; Gustavo.FernandezRomero@tuhs.temple.edu (G.F.-R.); gerard.criner@tuhs.temple.edu (G.J.C.); 10 Research Center on Asthma and COPD, Faculty of Medical Sciences, University of Ferrara, 44121 Ferrara, Italy; ppa@unife.it; 11 Institute of Clinical Medicine, Faculty of Health Sciences, University of Copenhagen, 1165 Copenhagen, Denmark

**Keywords:** chronic obstructive pulmonary disease, COPD, COPD exacerbations, outcomes, endpoints, treatment success, cure, randomised controlled trials, systematic reviews, clinical trials methods

## Abstract

A recently published ERS core outcome set recommends that all trials of COPD exacerbation management should assess the treatment success (or “cure” of the exacerbation), defined as a dichotomous measure of the overall outcome of an exacerbation. This methodological systematic review describes and compares the instruments that were used to assess treatment success or failure in 54 such RCTs, published between 2006–2020. Twenty-three RCTs used composite measures consisting of several undesirable outcomes of an exacerbation, together defining an overall unfavourable outcome, to define treatment failure. Thirty-four RCTs used descriptive instruments that used qualitative or semi-quantitative descriptions to define cure, marked improvement, improvement of the exacerbation, or treatment failure. Treatment success and failure rates among patients receiving guidelines-directed treatments at different settings and timepoints are described and could be used to inform power calculations in future trials. Descriptive instruments appeared more sensitive to treatment effects compared to composite instruments. Further methodological studies are needed to optimise the evaluation of treatment success/failure. In the meantime, based on the findings of this systematic review, the ERS core outcome set recommends that cure should be defined as sufficient improvement of the signs and symptoms of the exacerbation such that no additional systemic treatments are required.

## 1. Introduction

While our understanding of the mechanisms of acute exacerbations of chronic obstructive pulmonary disease (COPD) is expanding, their management has remained suboptimal and unchanged for many years [1,2]. Therefore, there is an urgent need to develop effective treatments and test them in high-quality randomised controlled trials (RCTs). The significant complexity and heterogeneity of COPD exacerbations has proven to be a substantial hindrance to the discovery of novel treatments. Nevertheless, the differential aetiology of exacerbations (e.g., events caused by bacterial or viral infections, or triggered by enhanced eosinophilic inflammation), is progressively being disentangled revealing targets for precision medicine interventions [3,4,5,6]. The clinical validation of biomarkers, such as procalcitonin or blood eosinophils, and aetiological classification of exacerbations may facilitate the future introduction of targeted treatments in clinical practice [3,4,5,6]. Precision medicine interventions for COPD exacerbations management are anticipated to be tested in rigorous RCTs in the coming years. The DECODE-NET (DisEntangling Chronic Obstructive Pulmonary Disease Exacerbations—an international clinical trials network) involving over 50 centres with expertise in COPD exacerbations trials research globally, is committed to conducting such RCTs [7].

RCTs on the management of COPD exacerbations are complicated both regarding design and conduct [7]. Different methodological aspects of such RCTs were evaluated in a recent systematic review, that revealed significant heterogeneity in the definition and diagnostic criteria of COPD exacerbations, as well as the outcomes (endpoints) evaluated across trials of COPD exacerbations management [8,9]. It is crucial that trials evaluating the management of a disease entity assess the same outcomes, those important to patients and health professionals, to facilitate decision making and improve the comparability of the trial results [10]. For this reason, the European Respiratory Society (ERS) launched a task force that developed a core outcome set for RCTs on the management of COPD exacerbations [11,12]. A core outcome set is an agreed standardised set of critically important outcomes that should be measured and reported as a minimum in all clinical trials in specific areas of health and health care [10].

The assessment of the overall outcome of the exacerbation (treatment success/failure, or cure) was selected as a core outcome, to be assessed in all future RCTs [12]. Moreover, a recent methodological systematic review revealed that this outcome is the second most frequently evaluated outcome in therapeutic trials of COPD exacerbations [9]. However, the definitions and instrument used to evaluate this outcome are very heterogeneous, limiting the interpretability and comparability of trial results. This meta-epidemiological study was conducted to inform the core outcome set and aimed to systematically evaluate the measurement instruments used for assessing treatment success or failure, to explore how effective they are, and which timepoints are more sensitive.

## 2. Materials and Methods

This meta-epidemiological study was based on a prospectively registered protocol (PROSPERO ID: CRD42020222287) [13]. For conducting and reporting this systematic review, we followed the standard methodology recommended by the Cochrane Collaboration [14] and the Preferred Reporting Items for Systematic Reviews and Meta-analyses (PRISMA) statement [15], respectively.

We systematically searched Medline/PubMed, the Cochrane Airways Trials Register [16], and the COSMIN (Consensus-based Standards for the Selection of Health Measurement Instruments) database on 12 November 2020, to identify trials testing pharmacological and non-pharmacological interventions for the management of COPD exacerbations. We also looked for methodological studies assessing the performance characteristics of different instruments for assessing treatment success or failure in clinical trials on COPD exacerbations. Detailed search strategies are available in Appendix A. Ongoing and completed trials and relevant methodological studies reported in the English language during the last 15 years (from 2006 onwards) were considered eligible. The titles and abstracts of all studies identified through the searches and the full texts of all potentially eligible studies were independently evaluated for eligibility by two review authors. We selected studies reporting on any of the following outcomes: cure, resolution, treatment success, treatment failure, time-to-cure, time-to-resolution, time-to-treatment success, or time-to-treatment failure. Relevant data on the design, interventions, baseline characteristics and imbalances, as well as data on the outcomes of interest, including the definitions used, measurement timepoints, and outcome data (findings) were extracted in a structured Excel form by one author and cross-checked by a second review author. The risk of methodological bias was assessed using the Cochrane Risk of Bias 1 tool by one author and cross-checked by a second author [17]. Disagreement in each stage of the process was resolved by consensus, involving a third author.

For the purposes of this review, we defined treatment success/failure, or cure of the exacerbation, as a dichotomous measure of the overall outcome of the exacerbation (Table 1). We excluded continuous measures evaluating changes in variables without prespecified thresholds of success or failure (e.g., change in symptom scores from baseline) and outcomes that did not focus on an overall assessment of the treatment outcome but on specific aspects of the exacerbation (e.g., death; admission to the intensive care unit; hospital admission; bacteriological eradication).

The definitions and timepoints of evaluation of the relevant outcomes were described narratively and in a tabulated format. Instruments used to measure the outcome of interest were grouped based on their characteristics into (i) composite instruments and (ii) descriptive instruments (definitions in Table 1). Grouping was based on consensus among the authors.

Treatment success or failure is a time-dependent outcome. Therefore, it is crucial to select the optimal timepoint for evaluating this outcome. For this reason, we explored the proportion of participants receiving usual care that fulfilled the criteria of treatment success or failure at different timepoints. Studies were stratified according to (i) the instrument used for assessing treatment success and (ii) the treatment setting that was considered to reflect the severity of the exacerbations. In this analysis, we included all treatment arms of the included trials in which participants received treatments that are consistent with international guideline recommendations (i.e., we excluded study arms that received novel experimental treatments).

Finally, to assess which instrument group and timepoints are more effective in identifying treatment effects, we explored between-group differences in treatment success or failure in trials assessing an intervention hypothesised by the trial investigators to be superior to the control group treatment (i.e., trials evaluating additional treatment compared to standard care; we excluded non-inferiority trials or trials comparing treatments without a prospective hypothesis around superiority). Outcome data from studies that were eligible for this analysis are presented in forest plots and described narratively.

## 3. Results

**Figure A1:**
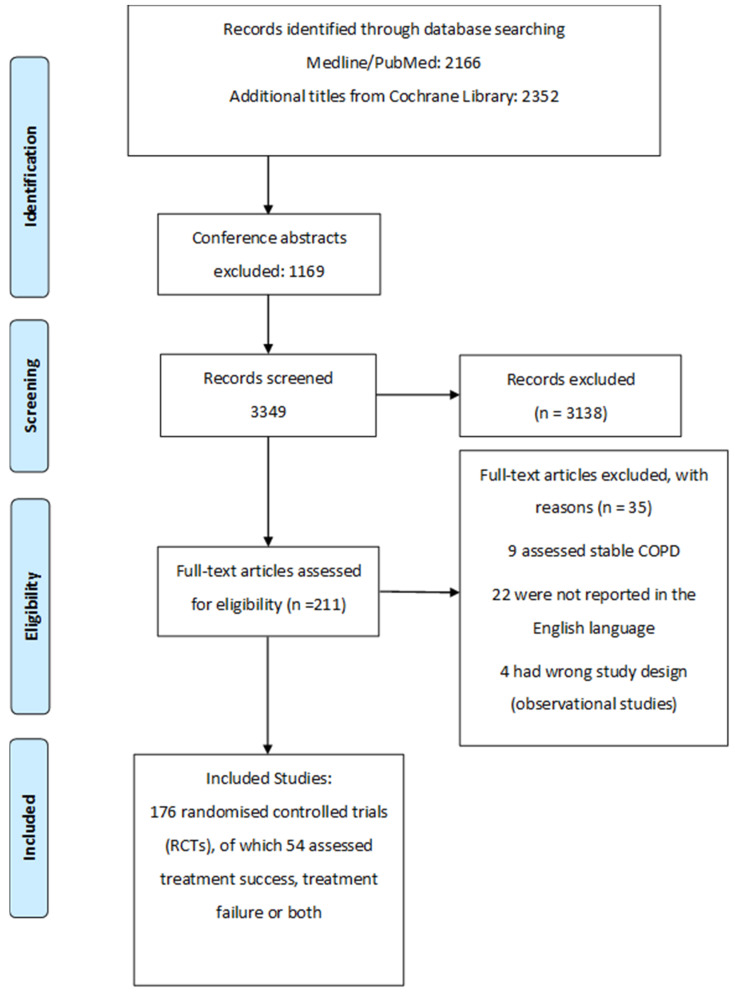
PRISMA Flowchart.

After removing duplicate records and conference abstracts, our searches yielded 3349 records. The selection process is described in a PRISMA diagram (Figure A1). We did not identify any eligible methodological studies evaluating the performance characteristics of instruments used to assess treatment success or failure in COPD exacerbations trials. We identified 176 ongoing or completed RCTs evaluating pharmacological or non-pharmacological interventions for the management of COPD exacerbations, of which 54 (30.7%) assessed the overall outcome of the index exacerbation (treatment success or treatment failure; references of all included studies are available in the online Appendix). This was selected as the primary outcome in 35 (64.8%), and as a secondary outcome in 19 (35.2%) of these trials. Timepoints of evaluation of this outcome varied from 2 h to 1 year after recruitment across the included trials. The interventions evaluated in the 54 included RCTs were antibiotics (*n* = 28), anti-inflammatories (11), oxygenation or non-invasive ventilation techniques (8), Chinese traditional medicine (3), or other interventions (4).

Two categories of outcome measurement instruments for evaluating treatment success or failure were revealed: composite and descriptive instruments (definitions in Table 1).

### 3.1. Composite Endpoints Consisting of Several Undesirable Outcomes of an Exacerbation

Twenty-three RCTs included 27 composite measurement instruments [4,18,19,20,21,22,23,24,25,26,27,28,29,30,31,32,33,34,35,36,37,38,39]. Most of these RCTs were at high or unclear risk of methodological bias. High risk of performance or detection bias was observed in 12/23 (52.2%) and 11/23 (47.8%) RCTs, respectively. Only six RCTs were deemed to be of an overall low risk of bias (Table 2).

Each composite instrument included a median of three (range 2–5) components. These components described different undesirable events and if any of these events was fulfilled then participants were considered to have experienced treatment failure. The most frequently used components were death (*n* = 16, 59.3% of the outcomes), need for hospital admission or re-admission (14, 51.9%), and treatment intensification (14, 51.9%). More details are summarised in Table 3.

### 3.2. Qualitative or Semi-Quantitative Descriptions of the Participants’ Clinical Status

Thirty-four RCTs included 45 descriptive instruments [37,38,39,40,41,42,43,44,45,46,47,48,49,50,51,52,53,54,55,56,57,58,59,60,61,62,63,64,65,66,67,68,69,70]. All but three trials were deemed to be at high risk of methodological bias. A high risk of performance or detection bias was revealed in 16 (47.1%) and 13 (38.2%) of the 34 studies, respectively (Table 4). Four states were defined: cure, marked improvement, improvement, and treatment failure. The definitions of these states differed across the included trials (Table 5). Moreover, the definition of clinical effectiveness varied. While in most trials, cure of the exacerbation or the absence of treatment failure was defined as treatment success, other trials accepted marked improvement, or, less frequently, improvement as an indicator of effectiveness (Table 5). The previous terms were used in many of the included trials. The instruments described in the remaining trials were matched to the most appropriate states by consensus among the investigators.

### 3.3. Proportion of Participants Experiencing Treatment Success or Failure over Time

Treatment success or failure is a time-sensitive outcome. Too early or too late during the exacerbation, nearly none or all the participants will have fulfilled the criteria of success or failure, respectively, limiting the ability of the outcome to detect between-group differences in clinical trials. For this reason, we explored the proportion of participants fulfilling the outcomes of interest at different timepoints.

**Figure 1:**
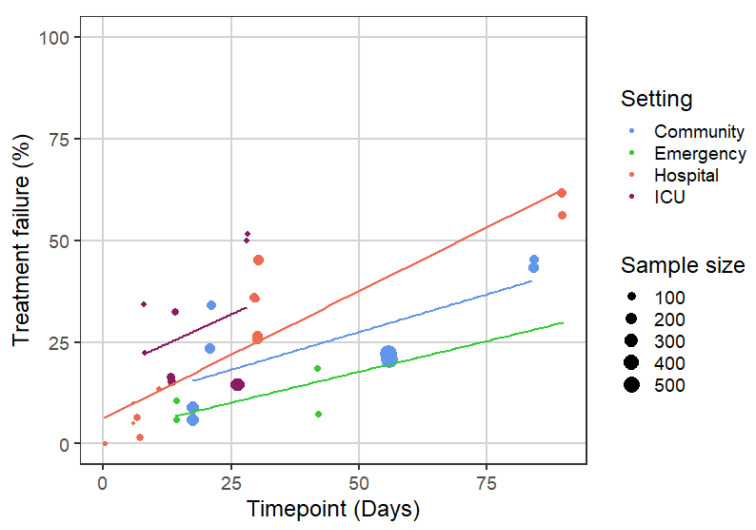
Treatment failure rates assessed using composite measurement instruments among participants in arms of the included trials that received treatments/interventions that are consistent with current clinical practice guidelines (i.e., study arms with experimental interventions that are not consistent with current clinical practice were excluded from this analysis).

Figure 1 depicts the proportion of study participants in treatment arms treated with guideline-recommended treatments (usual care) that experienced treatment failure as judged by composite outcome measurement endpoints (defined based on several undesirable outcomes of an exacerbation). This outcome was assessed at different timepoints, mostly within a month from recruitment, although in some trials it was tested at up to three months follow-up (and in one case at 9 months; not included in Figure 1).

The proportion of participants experiencing treatment failure based on these outcomes increased over time, as all participants fulfilling the criteria of treatment failure at any time until the selected timepoint were considered to have experienced the outcome (treatment failure). Importantly, treatment failure assessed at a later follow-up usually also included patients experiencing a re-exacerbation. As anticipated, treatment failure rates and slopes over time were higher among people admitted to the hospital or treated in the intensive care unit (ICU). When assessed between one and two weeks from recruitment, the median (range) of the treatment failure rates across the included studies were 8.3% (6–10.6%) in the emergency setting, 6.5% (1.5–13.5%) in the hospital setting, and 19.3% (15.3–34.2%) in the ICU setting. At three months follow-up, in studies conducted in the hospital setting, over half of the participants were identified as having experienced treatment failure. Moreover, 40% of participants treated in the community and 30% of those assessed in the emergency department were also anticipated to have experienced treatment failure at three months.

**Figure 2:**
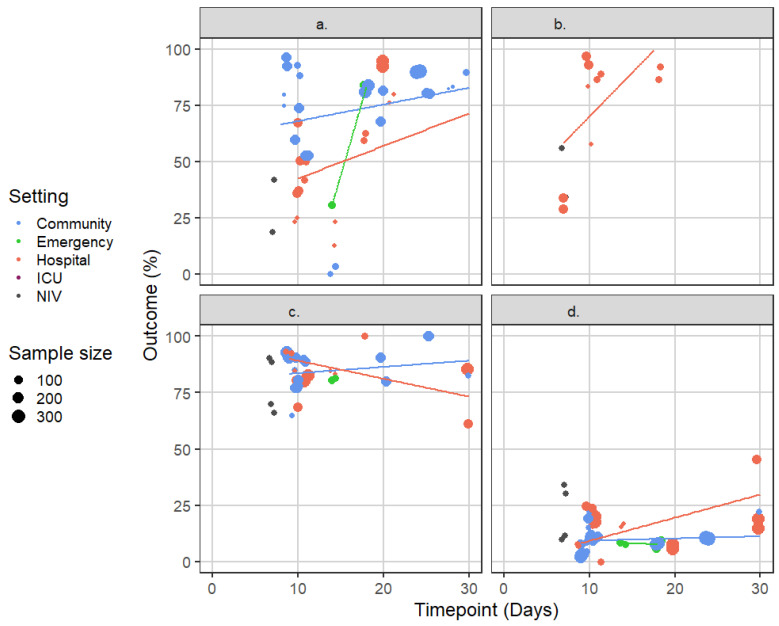
(**a**) Cure, (**b**) marked improvement, (**c**) improvement, or (**d**) treatment failure assessed using descriptive measurement instruments among participants in arms of the included trials that received treatments/interventions that are consistent with current clinical practice guidelines (i.e., study arms with experimental interventions that are not consistent with current clinical practice were excluded from this analysis).

The proportions of study participants fulfilling descriptive criteria for (a) cure, (b) marked improvement, (c) improvement, or (d) treatment failure, at different timepoints, are summarised in Figure 2. These states were evaluated at different timepoints, up to one month from recruitment, except for two studies that assessed cure or treatment failure at three months (not depicted in Figure 2).

When assessed between one and two weeks from recruitment, the median (range) of cure rates across the included studies were 74.5% (0–96.5%) in the community setting, 30.6% (30.5–30.7%) in the emergency setting, 36.4% (12.5–67.2%) in the hospital setting, and 30.2% (18.6–41.9%) in the NIV setting. The median (range) for marked improvement were 85.0% (28.9–96.9%) in the hospital and 45.1% (34.1–56.1%) in the NIV setting. The respective figures for improvement were 85.1% (64.9–92.8%) in the community, 81% (80.6–81.5%) in the emergency, 84.6% (68.6–100%) in the hospital, and 79.1% (65.9–90.2%) in the NIV settings. Finally, treatment failure rates were 10.0% (1.8–22.0%) in the community, 8.0% (7.7–8.3%) in the emergency, 15.4% (0–24.4%) in the hospital, and 20.9% (9.8- 34.1%) in the NIV settings.

Overall, the proportion of participants experiencing cure or marked improvement varied significantly during the first two weeks of follow-up, largely due to the significant variability in the outcome definitions. Stricter instruments, such as those requiring a complete resolution of all signs and symptoms associated with the exacerbation to confirm cure yielded lower cure rates, while higher rates were observed with more lenient definitions. Most of the included studies assessed patients treated in the community, or in the hospital for their exacerbation. As anticipated, cure rates were generally higher among participants treated in the community compared to those hospitalised, for any given follow-up timepoint.

The proportion of participants experiencing improvement or treatment failure varied less across the included studies and was less dependent on the instruments or timepoints of evaluation.

### 3.4. Measurement Timepoints and Treatment Effects

**Figure 3:**
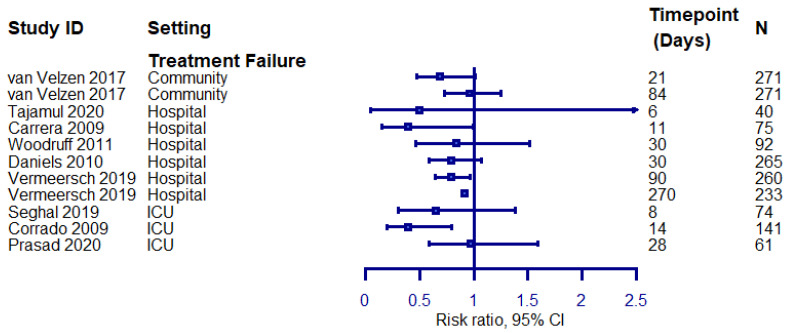
Treatment effects on treatment failure rates in superiority trials assessing treatment failure as a composite outcome. The left-hand side favours the intervention. N: study population.

**Figure 4:**
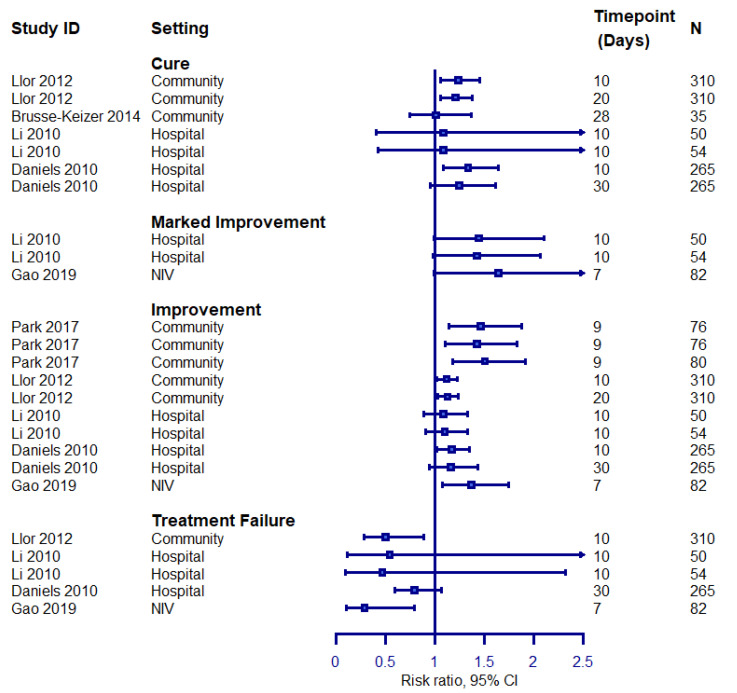
Treatment effects on treatment failure rates in superiority trials assessing cure, marked improvement, improvement, or treatment failure defined using descriptive instruments. The left-hand side favours the intervention. N: study population.

Finally, we explored treatment effects observed on the overall outcome of the exacerbations in superiority trials comparing an intervention hypothesised to be superior to the control group treatment by the trial investigators. Our aim was to explore whether specific instruments or measurement timepoints are more likely to yield a positive result. Forest plots summarising the findings from eligible outcomes are presented in Figure 3 and Figure 4.

Composite treatment failure outcomes appear to infrequently yield significant results (3/11, 27% of the evaluated outcomes; it should be noted that two of the three outcomes revealing a positive effect among hospitalised patients represent different timepoints from the same trial). We did not observe an association between specific measurement instruments or timepoints and positive treatment effects.

Over half of the outcomes evaluating cure or improvement yielded significant results, while 40% of those assessing treatment failure using descriptive instruments also yielded significant results. Nonetheless, the main difference between outcomes yielding positive or negative results was the study population of the included studies, rather than the measurement instruments or timepoints. Only two studies included in this analysis evaluated marked improvement, and the lack of any positive treatment effects most likely reflects the limited study population included in the respective analyses.

## 4. Discussion

This methodological systematic review evaluated the instruments used to assess treatment success or failure in RCTs of COPD exacerbations management and the timepoints in which these outcomes are measured. We found substantial heterogeneity in both the instruments and timepoints, which could significantly hinder the interpretability and comparability of the trial results. We identified two broad groups of measurement instruments assessing treatment success or failure: (i) composite outcomes consisting of several undesirable outcomes of exacerbations, together defining an overall unfavourable outcome; and (ii) instruments defining treatment success or failure based on qualitative or semi-quantitative descriptions of the patients’ clinical status with regards to their exacerbation. We present the rate of participants anticipated to experience treatment success or failure at different timepoints after recruitment in different trial settings, and these figures could be used to inform power calculations for future trials. Available data from eligible studies did not suffice to identify an optimal instrument or timepoint for evaluating treatment success or failure in COPD exacerbations.

Composite treatment failure measurement instruments are characterised by a critical drawback. They group together components that bear a very different impact (utility) on patients, such as death versus the need for supplement oxygen [71]. Importantly, the relative frequency of these outcomes may vary across the different exacerbation subtypes, thus limiting the interpretability of the results. For example, exacerbations caused by a bacterial infection are associated with higher mortality, while an increased re-hospitalisation rate is observed in exacerbations characterised by enhanced eosinophilic inflammation [2,72]. Moreover, our analysis suggests that composite instruments are less sensitive in identifying treatment effects compared to descriptive instruments, as fewer studies using composite instruments identified a statistically significant effect in trials evaluating interventions that the investigators hypothesised were superior to the control treatments. While this finding is indirect and based on a small number of observations, it may reflect a limited sensitivity of these instruments. Finally, the ERS COPD exacerbations core outcome set recommends that most of the components of these composite outcomes should be assessed as independent outcomes, thus providing additional granularity in the trial results, while in parallel limiting the utility of composite instruments assessing treatment success or failure [12].

More trials used descriptive instruments for assessing the overall outcome of exacerbations. These instruments are limited by the subjectivity of assessing the severity of the clinical conditions by patients and clinicians alike. As a result, these instruments may be susceptible to performance and detection bias. A similar limitation is accepted in the methodology used to classify exacerbations by severity, depending on the clinician’s judgement around the need for systemic treatments or hospital admission [8,73]. These problems spring from the significant heterogeneity that characterises acute exacerbations of COPD and from the lack of clinically validated clinical biomarkers or objective indices, that could facilitate severity assessment or confirmation of cure.

In the absence of adequate data to select an optimal measurement instrument for assessing the cure of an exacerbation, the ERS COPD exacerbations core outcome set panel recommended an interim instrument for evaluating this outcome based on the evidence-informed consensus among the panel members and participating patient representatives. It is recommended that treatment success (cure) should be defined as the sufficient improvement of the signs and symptoms of the exacerbation such that no additional systemic treatments (systemic corticosteroids or antibiotics) are required [12]. This instrument aligns with the definitions of COPD exacerbations severity and is a practical outcome that is routinely considered in daily clinical practice and often used in trials. In parallel, the panel issued a recommendation for methodological research to develop objective and accurate methods for confirming the cure of COPD exacerbations.

The most frequently used descriptive instrument defined cure as complete resolution of all signs and symptoms of an exacerbation. However, this instrument was not adopted in the core outcome set due to limitations that may have limited its usability. Firstly, large observational studies have demonstrated that the recovery period of an exacerbation varies and may be very prolonged [74,75]. It has been suggested that one in four patients experience persistent symptoms compared to their pre-exacerbation status in excess of 25–35 days after the exacerbation’s onset [74,75], while recovery of the patient’s pre-exacerbation exercise capacity or activities of daily living may be further delayed [76,77]. Moreover, acute exacerbations expedite the progression of COPD. As a result, the clinical condition of patients after recovery from an exacerbation may be characterised by a greater symptomatic burden compared to the previous baseline [77]. Therefore, anticipating the complete resolution of all signs and symptoms caused by the exacerbation may not be appropriate; in addition, this outcome may be more susceptible to subjectivity in the assessment of the potentially limited and clinically insignificant remaining symptoms during recovery.

Another interesting instrument defined treatment success as the first of three days while patients’ symptoms are back at their baseline, or the first of seven days in which patients only report a minor increase in symptoms compared to baseline, without fever or change in sputum colour. This instrument has only been used in a limited number of trials and is not adequately validated, and for this reason it was not adopted by the core outcome set panel. However, this outcome may provide more consistency and allow trialists to measure more accurately time-to-treatment success. Therefore, it may be worth being further validated in future trials.

There is significant variability in the terms used to describe treatment success, such as cure, resolution, or remission. Cure may be a confusing term, since COPD is a chronic, incurable disease. On the other hand, remission is associated with negative connotations (cancer). Treatment success or resolution are more appropriate terms.

Treatment success or failure is frequently evaluated as an outcome in other acute respiratory diseases as well, including community, hospital-acquired, or ventilator-associated pneumonia, COVID-19, and acute asthma [78,79] [unpublished data]. Trialists face similar challenges in the selection of appropriate instruments for evaluating this outcome in these acute respiratory diseases [78] [unpublished data]. We were not able to identify any other methodological studies evaluating instruments for measuring treatment success or failure in any acute respiratory diseases.

As previously mentioned, the course and outcomes, but also treatment responses of different COPD exacerbation subtypes, such as those caused by bacterial or viral infections, or those characterised by enhanced eosinophilic inflammation, vary significantly [2,72,80,81]. Clinical trialists should consider conducting more personalised trials, focusing on specific exacerbation subtypes, as the study populations, treatment effects, and outcomes would be more homogeneous and more easily interpretable. Current data strongly suggest that the therapeutics of COPD exacerbations will progress through precision medicine approaches [2,82].

This meta-epidemiological study was limited by the inadequate number of included RCTs and was therefore not able to identify an optimal instrument and timepoints for assessing treatment success in clinical trials in COPD exacerbations. We only included trials published from 2006 onwards. However, we considered that the inclusion of older trials might have introduced heterogeneity in our findings, as the diagnosis, severity stratification, and management of exacerbations may have differed in studies conducted previously. Similarly, clinical trial methodology has changed over the last decades and so has our approach towards trial outcomes. Moreover, we did not include data from observational studies, as our work focuses on clinical trials and the instruments used in observational studies are often different.

The thorough systematic search, which included the Cochrane Airways Trials Register, sourcing clinical trials from five electronic databases, and the abstract proceedings of all major international respiratory conferences, is one of the strengths of this study. Another major strength is the thorough analysis of the instruments used to assess treatment failure, the timepoints at which they were evaluated, and the results they yielded. Finally, the last part of this manuscript was informed by the consensus discussions of a multi-stakeholder panel with a global representation, described in the main task force report [12].

## 5. Conclusions

Various instruments and timepoints are currently used to assess treatment failure in clinical trials evaluating COPD exacerbation management. Further methodological studies are needed to identify the optimal instrument. In the meantime, in line with the ERS COPD exacerbations core outcome set, we recommend that COPD trials should evaluate treatment success based on the need for additional systemic treatments after the completion of the initial treatment of the exacerbation.

## Figures and Tables

**Table 1:** Definitions of treatment success/failure and of the measurement instruments’ classification.

Term	Definition
Treatment success/failure, or cure	A dichotomous measure of the overall outcome of the exacerbation. We excluded continuous measures evaluating a change in variables without prespecified thresholds of success or failure (e.g., change in symptom scores from baseline) and outcomes that did not focus on an overall assessment of the treatment outcome but on specific aspects of the exacerbation (e.g., death; hospital admission; bacteriological eradication).
Composite instruments	Instruments consisting of several undesirable outcomes of an exacerbation (e.g., death; need for treatment intensification; admission to the intensive care; or hospital admission), together defining an overall unfavourable outcome.
Descriptive instruments	Instruments defining treatment success or failure based on qualitative or semi-quantitative descriptions of the patients’ clinical status with regards to the exacerbation at a specific timepoint. The following states are often defined: cure, marked improvement, improvement, or treatment failure.

**Table 2:** Risk of bias of RCTs reporting composite outcome measurement instruments.

	Sequence	Allocation	Performance	Detection	Attrition	Reporting	Other
Aaron 2013	Low	Low	Low	Low	Low	Low	Low
Aggarwal 2011	Low	Unclear	High	High	Low	Unclear	Unclear
Bafadhel 2012	Low	Low	Low	Low	Low	Low	Low
Carrera 2009	Low	Low	Low	Low	High	Unclear	Low
Corrado 2009	Low	Low	High	High	Low	Unclear	Unclear
Daniels 2010	Low	Low	Low	Low	Low	High	Low
de Jong 2007	Low	Unclear	Low	Low	Low	Low	Low
Goossens 2013	Low	Low	High	High	Low	Low	Low
Hua 2020	Low	Low	High	High			
Jolliet 2016	Low	Unclear	High	Low	Low	Low	Low
Nicolini 2014	Low	Low	High	High	Low	Low	Low
Nouira 2010	Low	Low	Low	Low	Low	Low	Low
Papalampidou 2020	Low	High	High	High			
Prasad 2020	Low	Low	High	High	Low	Low	High
Sehgal 2019	Low	Low	High	High	Low	Low	Low
Sivapalan 2019	Low	Low	High	High	Low	Low	Low
Strambu 2019	Low	Low	Low	Low	Low	Low	Low
Tajamul 2020	Low	Low	High	High	Low	Low	Low
Urueta-Robledo 2006	Unclear	Unclear	Low	Low	High	Unclear	Unclear
van Velzen 2017	Low	Low	Low	Low	Low	Low	Low
van Zanten 2007	Unclear	Unclear	High	High	Low	Unclear	Low
Vermeersch 2019	Low	Low	Low	Low	Low	Low	Low
Wilson 2015	Unclear	Unclear	Low	Low	High	Low	Low
Woodruff 2011	Unclear	Unclear	Low	Low	High	Low	High

**Table 3:** Undesirable outcomes included in the composite treatment failure instruments, along with the frequency in which they were utilised.

Components of the Composite Outcome Definitions	N (%)
Death	16 (59.3%)
Need for hospital admission/re-admission	14 (51.9%)
Need for treatment intensification	14 (51.9%)
Need for endotracheal intubation/mechanical ventilation	10 (37.0%)
Persistent or deteriorating symptoms and signs	8 (29.6%)
Need for non-invasive ventilation	3 (11.1%)
Need for urgent outpatient or emergency room visit	3 (11.1%)
New infection	3 (11.1%)
Need for higher level of hospital care	2 (7.4%)
Deteriorated arterial blood gases	1 (3.7%)
Hemodynamic instability	1 (3.7%)
Need for ICU admission	1 (3.7%)
Prolonged hospital stay	1 (3.7%)
Reduced level of consciousness	1 (3.7%)
Treatment intolerance	1 (3.7%)

**Table 4:** Risk of bias of RCTs reporting descriptive instruments.

	Sequence	Allocation	Performance	Detection	Attrition	Reporting	Other
Alvarez-Sala 2006	Unclear	Unclear	Low	Low	Low	Unclear	Unclear
Andre-Alves 2007	Unclear	Unclear	High	High	Low	Unclear	High
Blasi 2013	Unclear	Unclear	High	High	Low	Low	Unclear
Blasi 2013 B	Low	Low	Low	Low	Low	Unclear	High
Brusse-Keizer 2014	Low	Low	Low	Low	Low	Low	High
Ceviker 2014	Low	Low	High	Low	High	Unclear	Unclear
Chatterjee 2011	Low	Low	High	Low	Low	Unclear	Unclear
Daniels 2010	Low	Low	Low	Low	Low	High	Low
Gao 2019	Unclear	Unclear	High	High	Low	Unclear	Low
Giusti 2016	Low	Low	Low	Low	Low	Low	Low
Gotfried 2007	Unclear	Unclear	High	High	Low	Low	Low
Jiang 2017	Low	Unclear	High	High	Low	Unclear	Low
Li 2010	Low	Unclear	High	High	High	Unclear	Low
Llor 2009	Unclear	Low	Low	Low	Low	Unclear	Low
Llor 2012	Low	Unclear	Low	Low	Low	Low	Low
Nouira 2010	Low	Low	Low	Low	Low	Low	Low
Park 2017	Unclear	Low	Low	Low	Low	Unclear	High
Petitpretz 2007	Unclear	Unclear	High	High	High	Unclear	Unclear
Prins 2019	Low	Low	High	High	Low	Low	Low
Rhee 2015	Low	Low	Low	Low	High	High	High
Ritchie 2019	Low	Low	Low	Low	Low	Low	Low
Roede 2007	Low	Low	Low	Low	Low	Unclear	High
Rohde 2015	Unclear	Unclear	Low	Low			
Stallberg 2009	Unclear	Low	Low	Low	Low	Low	Low
Stolz 2007	Unclear	Unclear	High	High	Low	Low	Low
Urueta-Robledo 2006	Unclear	Unclear	Low	Low	High	Unclear	Unclear
van den Broek 2008	Low	Low	Low	Low	Low	Unclear	Unclear
van Zanten 2007	Unclear	Unclear	High	High	Low	Unclear	Low
Verduri 2015	Low	Low	High	High	Low	Low	Low
Wang 2010	Unclear	Low	Low	Low	Low	High	Unclear
Xie 2019	Low	Low	High	Low			
Yoon 2013	Low	Low	High	High	Low	Unclear	Unclear
Zervos 2007	Unclear	Unclear	High	High	Low	Unclear	High
Zhang 2019	Low	Low	Low	Low			

**Table 5:** Definitions of various COPD exacerbation states within descriptive instruments.

COPD Exacerbation States Described	N
**Cure or Resolution**	
✓ Complete resolution of all signs and symptoms of the exacerbation.	8
✓ Sufficient improvement of the signs and symptoms such that no additional systemic treatments were prescribed.	5
✓ Anthonisen Respiratory Symptoms Score < 5 [46].	2
✓ Three consecutive days when patients’ symptoms were back at their baseline, or seven consecutive days in which the patient only reported a “minor increase” in symptoms compared to baseline, without fever or change in sputum colour.	2
✓ Resolution of symptoms, signs, and laboratory findings.	1
✓ Resolution of symptoms, signs, laboratory findings, and eradication of the causative organism.	1
✓ Remission (not further described).	4
**Marked Improvement**	
✓ Resolution of all signs and symptoms of the exacerbation, or reduction of at least 3 points in a non-validated score, compared to baseline.	2
✓ Only one of the following parameters remains abnormal: clinical symptoms, signs, laboratory findings, causative pathogen (not eradicated).	1
✓ Major symptoms including cough, exacerbation, and dyspnoea almost disappeared and the chest imaging was significantly improved.	1
✓ Significantly improved symptoms, signs, and laboratory tests. Effectiveness index between 60–90% (based on a non-validated scale).	1
**Improvement**	
✓ Improved signs and symptoms, without any new signs or symptoms.	4
✓ Improved symptoms as evaluated by clinical scores: Anthonisen Respiratory Symptoms Score between 6–10; 30% improvement in the Bronchitis Severity Score (BSS); reduction of 1–3 points in a non-validated score.	3
✓ Improved, but more than one of the following parameters remain abnormal: clinical symptoms, signs, laboratory findings, causative pathogen (not eradicated).	1
✓ Improved symptoms, signs, and laboratory tests. Effectiveness index between 30–60% (based on a non-validated scale).	1
✓ Resolution of at least 50% of symptoms back to the baseline level.	1
✓ Resolution of fever with incomplete resolution of signs and symptoms, without the need for additional antibiotics.	1
✓ Resolution or reduction of the symptoms and signs without new symptoms and signs associated with the infection.	1
✓ Improvement (not further described).	4
**Treatment Failure**	
✓ Lack of resolution of signs and symptoms, requiring additional treatment, (or death).	8
✓ Persistence or worsening of signs and symptoms, or death.	7
✓ Lack of resolution of signs and symptoms or need for further treatment.	4
✓ Persistence or worsening of signs, symptoms, or laboratory tests.	1
✓ Worsening of at least one symptom, or no change in the symptoms, or reduction of less than 3 points in a non-validated score, compared to baseline.	1
✓ Ineffective treatment (not further described).	3

## Data Availability

Not applicable. No original data were collected as part of this systematic review and meta-epidemiological study.

## † DECODE-NET Collaborators

Shawn Aaron, Fekri Abroug, Alvar Agusti, Sachin Ananth, Amy Attaway, Nawar Bakerly, Per Bakke, Arianne Baldomero, Igor Barjaktarevic, Konstantinos Bartziokas, Bianca Beghe, Surya P Bhat, Andras Bikov, Joanne Billings, Charlotte Bolton, Arnaud Bourdin, Thomas Bradbury, Guy Brusselle, Robert Burkes, Cordula Cadus, Steven Cassady, Marco Contoli, Alexandru Corlateanu, Olga Corlateanu, Gerard Criner, Balázs Csoma, Victoria Dean, Benedicte Demeyere, Philip Diaz, Claudia C Dobler, Mark T Dransfield, Sean Duffy, Carlos Echevarria, Josephin Eklöf, Alexander Emelyanov, Rosa Faner, Gustavo Fernandez-Romero, Marie Fisk, Nate Gaeckle, Andrew Gangemi, Georgia Gkrepi, Zeineb Hammouda, MeiLan K Han, Nicholas S Hill, Peter Horvath, Arturo Huerta, John Hurst, Anand Iyer, Michael Jacobs, Christine Jenkins, Jens-Ulrik Jensen, Guy Joos, Elena Katibnikova, Olga Kharevich, Konstantinos Kostikas, Jerry A Krishnan, Ken Kunisaki, Christos Kyriakopoulos, Wassim Labaki, Stephanie LaBedz, David Lafon, Lies Lahousse, Elena Lapteva, Zsofia Lazar, Jörg Leuppi, Carol Liddle, John Linnell, Alejandra Lopez-Giraldo, Neil MacIntyre, Nathaniel Marchetti, Fernando D Martinez, Fernando J Martinez, Alexander G Mathioudakis, Vanessa McDonald, Rebecca McLoughlin, Anne Melzer, Takudzwa Mkorombindo, Christopher Mosher, Marilyn Moy, Mulpuru Sunita, Rune Nielsen, Ralph Panos, Alberto Papi, Trisha Parekh, Ian Pavord, Victor Pinto-Plata, Dimitris Potonos, Jennifer Quint, Robert M Reed, Jean Rommes, Isabel Saraiva, Galina Sargeeva, Frank C Sciurba, Dave Singh, Pradeesh Sivapalan, Nestor Soler, Jennifer So, Ecaterina Stavila, Daiana Stolz, Charlie B Strange, Carey Suehs, Fransesca Torsani, Jørgen Vestbo, Hao Wang, James Wells, Fuqiang Wen, Chris Wendt, Paula Williamson, Janelle Yorke, Massa Zantah.

## Appendix A

**Search strategy**

**Chronic Obstructive Pulmonary Disease:**

#1 Chronic Obstructive Pulmonary Disease [MH]
#2 Lung Diseases, Obstructive [MH: NOEXP]
#3 Emphysema [MH]
#4 Chronic Bronchitis [MH]
#5 COPD [tiab]
#6 COAD [tiab]
#7 “Chronic Bronchitis” [tiab]
#8 Emphysema [tiab]
#9 Obstructive [ti]
#10 (Pulmonary OR Respiratory OR Airway OR Airflow OR Lung) [ti]
#11 #9 AND #10
#12 #1 OR #2 OR #3 OR #4 OR #5 OR #6 OR #7 OR #8 OR #11

**Disease exacerbations:**

#13 Disease Exacerbation [MH]
#14 Exacerbation [tiab]
#15 Exacerbation* [tiab]
#16 #13 OR #14 OR #15

**RCT filter:**

#17 randomized controlled trial [pt]
#18 controlled clinical trial [pt]
#19 randomized [tiab]
#20 placebo [tiab]
#21 clinical trials as topic [mesh: noexp]
#22 randomly [tiab]
#23 trial [ti]
#24 #17 OR #18 OR #19 OR #20 OR #21 OR #22 OR #23

**SR filter:**

#25 Medline [tiab]
#26 Systematic [tiab] and (review [tiab])
#27 Meta analysis [publication type]
#28 Meta-analysis [tiab]
#129 Metaanalysis [tiab]
#30 #25 OR #26 OR #27 OR #28 OR #29

#31 Search (“2006” [Date-Publication]: “2020” [Date-Publication])
#32 animals [mh] NOT humans [mh]
#33 #12 AND #16 AND #31 AND (#24 OR #30)
#34 #33 NOT #32
